# Evolution of the Electronic Properties of Tellurium Crystals with Plasma Irradiation Treatment

**DOI:** 10.3390/nano14090750

**Published:** 2024-04-25

**Authors:** Congzhi Bi, Tianyu Wu, Jingjing Shao, Pengtao Jing, Hai Xu, Jilian Xu, Wenxi Guo, Yufei Liu, Da Zhan

**Affiliations:** 1 Department of Physics, College of Physical Science and Technology, Research Institution for Biomimetics and Soft Matter, Xiamen University, Xiamen 361005, China; 19820211153715@stu.xmu.edu.cn; 2 State Key Laboratory of Luminescence and Applications, Changchun Institute of Optics, Fine Mechanics and Physics, Chinese Academy of Sciences, Changchun 130033, China; wutianyu0308@126.com (T.W.); shaojingjing20@mails.ucas.ac.cn (J.S.); jingpt@ciomp.ac.cn (P.J.); xuhai@ciomp.ac.cn (H.X.); xujl@ciomp.ac.cn (J.X.); 3 College of Science, Beihua University, Jilin 132000, China; 4 Key Laboratory of Optoelectronic Technology & Systems, Chongqing University, Chongqing 400044, China

**Keywords:** tellurium crystal flake, plasma irradiation, tunnelling current, conductive atomic force microscopy (CAFM), band gap

## Abstract

Tellurium exhibits exceptional intrinsic electronic properties. However, investigations into the modulation of tellurium’s electronic properties through physical modification are notably scarce. Here, we present a comprehensive study focused on the evolution of the electronic properties of tellurium crystal flakes under plasma irradiation treatment by employing conductive atomic force microscopy and Raman spectroscopy. The plasma-treated tellurium experienced a process of defect generation through lattice breaking. Prior to the degradation of electronic transport performance due to plasma irradiation treatment, we made a remarkable observation: in the low-energy region of hydrogen plasma-treated tellurium, a notable enhancement in conductivity was unexpectedly detected. The mechanism underlying this enhancement in electronic transport performance was thoroughly elucidated by comparing it with the electronic structure induced by argon plasma irradiation. This study not only fundamentally uncovers the effects of plasma irradiation on tellurium crystal flakes but also unearths an unprecedented trend of enhanced electronic transport performance at low irradiation energies when utilizing hydrogen plasma. This abnormal trend bears significant implications for guiding the prospective application of tellurium-based 2D materials in the realm of electronic devices.

## 1. Introduction

Two-dimensional (2D) single-crystal materials have many applications in the fields of electronics, optics, magnetism, and other fields due to their quantum confinement effect in z-space [1,2,3,4,5,6]. The exotic properties of 2D materials have attracted tremendous attention in the scientific research community in recent years, especially in the field of electronics. Compared with traditional semiconductor materials consisting of silicon, most 2D materials have some shortcomings for their intrinsic electronic properties. Graphene cannot be used as an ideal logic device with high current switching ratios due to its zero band gap [2]; on the other hand, most transition metal chalcogenide compounds with appropriate natural band gaps can have high current switching ratios in logic devices, but they struggle to achieve high mobility [7]. Thus, 2D materials that simultaneously meet the characteristics of high current on–off ratio and high mobility have been one of the key focuses of research in 2D electronic materials in recent years. Among them, tellurium, a natural single-element-based 2D material with an appropriate band gap, has been demonstrated to present excellent intrinsic electronic properties with a high current switching ratio (~ 10^6^) and high carrier mobility (~700 cm^2^V^−1^s^−1^) [8,9,10,11], and it also shows relatively good stability in ambient air at room temperature. The band gap is ~1 eV and 0.35 eV for its monolayer and bulk, respectively [10,12]. Bulk tellurium can be fabricated into crystals through methods such as the hydrothermal approach [10], chemical vapor deposition (CVD) [13], and physical vapor deposition (PVD) [14]. Tellurium has a wide range of potential applications such as field effect devices [10,15], infrared devices [16,17], photodetectors [16,18,19], and piezoelectric devices [20,21]. Although tellurium possesses excellent intrinsic electronic properties, there is a relative lack of research on the stability of its own crystal structure and electronic structure modulation beyond its intrinsic electronic properties. Through physical modification, especially the method of plasma-based surface treatment, one can not only study the stability of its crystal structure but also reveal the evolution of electronic properties. Previously, plasma treatment has been commonly used to modify the crystal structure and electronic properties of 2D materials for both fundamental studies and practical applications [22,23,24,25]. Recently, plasma irradiation has shown great potential in preparing micro/nanostructures and optimizing material properties [26,27].

For tellurium, plasma irradiation-related papers are rare. In this work, the surface structure modification of tellurium crystal flakes was achieved through hydrogen plasma (HP) and Ar plasma (AP) irradiation treatments, respectively. Using the contact mode of conductive atomic force microscopy (CAFM) [28], the evolution of the electronic properties of the plasma-irradiated tellurium, corresponding to its surface structure modification, was revealed by scanning the contact tunnelling current properties biased between a conductive tip and the tellurium flakes. Combined with Raman spectroscopy analysis, it was confirmed that the tellurium crystal flakes did not undergo a phase transformation or chemical change during the plasma irradiation treatment, but the crystal structure would be gradually broken under a certain level of energy irradiation. It was also found in this work that through HP treatment, the low-irradiation-energy region can be used to improve the transport performance of tellurium crystal, but no corresponding improvement window was found for AP treatment.

## 2. Materials and Methods

### 2.1. Preparation of Tellurium Flakes

In a typical synthesis case, 100 mg of sodium tellurite powder and 498 mg of polyvinylpyrrolidone powder were weighed, and then they were added to 33 mL of deionized water, 1 mL of hydrazine hydrate, and 1 mL of ammonia aqueous solution, respectively. After fully stirring, the mixed solution was added to an autoclave reactor to keep it at 180 °C for 40 h. The reaction resulted in a solution of tellurium flakes, which was added to a centrifuge tube for repeated centrifugation. After ultrasonic washing to remove surface impurities, the remaining solution was filtered to obtain a relatively pure sample of tellurium flakes.

### 2.2. Preparation of Tellurium Devices to Be Tested

To isolate a single tellurium flake from the bulk material, adhesive tape was used to gently peel off the solid tellurium flakes collected by filtration. For the preparation of the conductive substrate, photolithography was employed followed by metal deposition methods to lay down a layer of gold (Au) thin film on the Si/SiO_2_ substrate. Utilizing a two-dimensional material transfer platform, the selected tellurium flake was carefully transferred onto the Au-coated Si/SiO_2_. After the transfer, the fabricated Si/SiO_2_/Au substrate with the tellurium flake was secured on a metal tray that was designed to fit into a conductive atomic force microscopy (CAFM) apparatus. Followed by that, an electrical connection between the Au film and the metal tray was established by applying conductive silver paste.

### 2.3. Plasma Treatment of Tellurium Flakes

The samples were irradiated by hydrogen and argon plasma using a Diener Zepto-BRS 200 plasma surface processor (Diener Electronic, Ebhausen, Germany). The instrument used a 13.56 MHz high-frequency current generator for discharge, with an input power of 100 W. The prepared sample was placed inside the vacuum chamber of the processor, maintaining the ventilation gas flow rate at 10 mL/min and keeping the chamber pressure at 0.1 mbar. Depending on the specific requirements of the follow-up experiments, a choice was made between hydrogen and argon for injection, both featuring a high purity level of 99.9%. The power and duration of the plasma irradiation increased gradually, with detailed parameters of the irradiation power and time provided in the Results section. The plasma irradiation-treated sample flakes were further characterized by conductive atomic force microscopy (CAFM), Raman spectroscopy, and optical microscopy, respectively.

## 3. Results and Discussion

### 3.1. Crystal Morphology and Band Gap Characterization for the Pristine Tellurium Flakes

**Figure 1:**
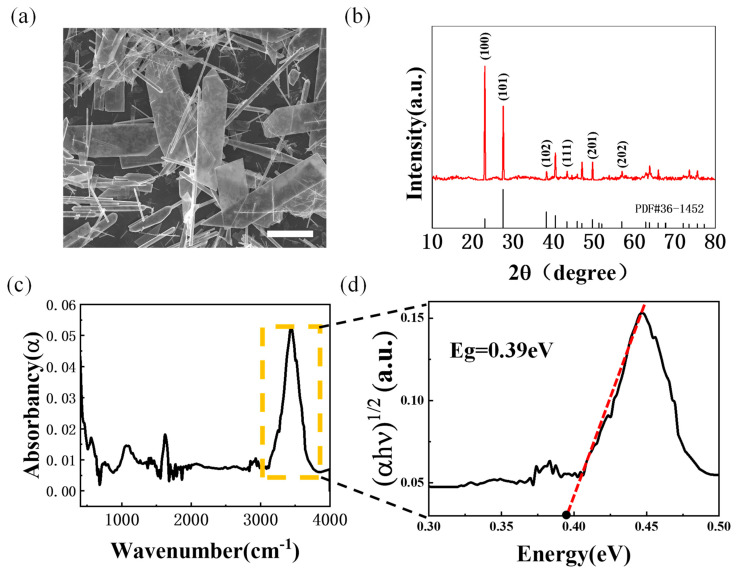
The characterization of tellurium flakes. (**a**) SEM image of tellurium flakes after hydrothermal processing, with scale bar of 5 μm. (**b**) XRD spectrum of tellurium flakes. (**c**) Infrared absorption spectrum of tellurium flakes. (**d**) The derived optical band gap spectra on the basis of the infrared absorption spectrum.

Tellurium powder with a nanoflake microstructure was prepared by a hydrothermal reaction according to a similar recipe to the method reported previously [10]. Figure 1a shows a scanning electron microscope (SEM) image of a tellurium sample obtained after hydrothermal processing. Most of the tellurium crystals are in the microstructural shape of rectangular sheets or rods stacked randomly on the substrate. The X-ray diffraction (XRD) results show a series of feature diffraction peaks for the tellurium sample, of which the positions of the peaks are completely consistent with the ideal tellurium structure (refer to the spectrum shown in Figure 1b as a comparison). Among them, the (100) and (101) diffraction peaks are particularly strong, and the full widths at half maximum of the peaks are as low as 0.22° and 0.21°, respectively, indicating that the synthesized crystals have good crystallinity. Based on the characterization of the crystal structure, the tellurium sample has obvious optical absorption feature peaks in the infrared region, as shown in Figure 1c. The corresponding optical band gap can be obtained through fitting on the basis of the Kubelka–Munk formula conversion [29,30]. The most prominent absorption peak shown in the yellow box of Figure 1c was chosen to be converted; as can be seen in Figure 1d, the optical band gap of tellurium is ~0.39 eV. The result is close to the reported band gap of bulk tellurium, indicating intrinsic electronic structure properties owing to the good crystalline quality.

### 3.2. Study of the Evolution in the Structure and Electronic Properties of Tellurium Crystal Flakes under Plasma Treatment

**Figure 2:**
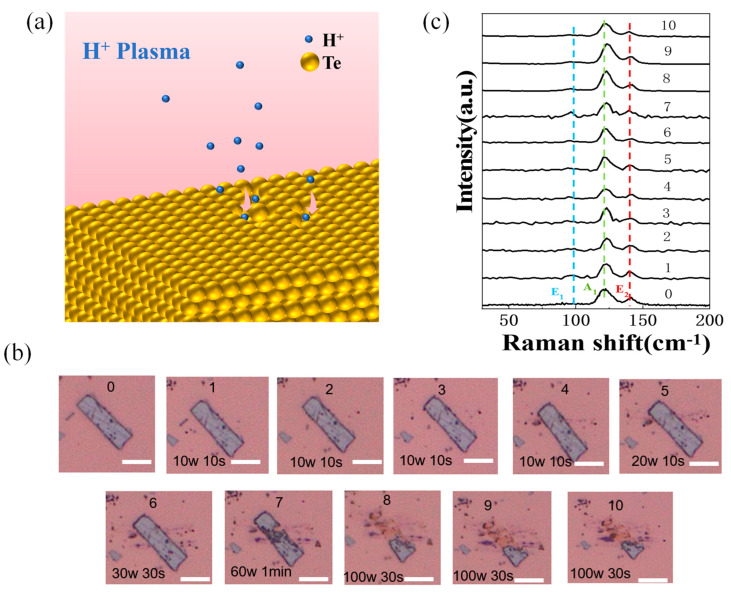
(**a**) Schematic diagram of the surface of tellurium crystal flake treated with HP. (**b**) Optical images of tellurium crystal flake successively treated with HP, with scale bar of 5 μm; the processing power and duration are marked accordingly. (**c**) Raman spectra of successive HP irradiation-treated tellurium crystal flake.

The HP-treated tellurium crystal flake surface is schematically shown in Figure 2a. The optical images were used to record the change in the tellurium crystal surface structure with gradually increased HP treatment power and duration (Figure 2b). For pristine tellurium before it underwent HP treatment, there are three Raman active modes that can be seen in the 0th curve in Figure 2c, which are the modes A_1_ (chain expansion in the ab plane), E_1_ (a- and b-axis rotation), and E_2_ (asymmetric stretching, mainly along the c-axis) [31]. At the beginning with relatively low HP irradiation power (10 W, 20 W, and 30 W), the crystal structure evolution does not show obvious changes compared to that of the pristine sample (see the optical images in Figure 2b 1–6). Each of the corresponding Raman spectra always clearly presents almost the same feature as that of the pristine tellurium (see the 1st–6th curves in Figure 2c), which illustrates that HP irradiation with low power and duration is “relatively” “gentle” for tellurium flakes. When the plasma irradiation power further increased to 60 W, the optical images show that the tellurium crystal started undergoing obvious structural damage on the surface after a total treatment duration of 90 s (see the optical image for the seventh treatment in Figure 2b). This obvious change may be due to the fact that the energy accumulation on the tellurium surface during HP treatment reached the crystal structure-breaking threshold. Therefore, once the irradiation power and energy exceed a certain value, the tellurium flake will undergo structural damage. HP treatment for the same tellurium sample under subsequent higher-power conditions (100 W) resulted in more obvious microstructural damage to the surface (see the optical images after the seventh treatment in Figure 2b). However, it was noted that no additional observable Raman peaks appeared in this sample, even when irradiated by HP with a power of 100 W and duration of 90 s (see the 8th–10th Raman spectra in Figure 2c). Fundamentally, we infer that the effect of HP on tellurium mainly causes microscopic crushing at the physical level but does not give rise to a structural phase transformation or introduce any chemical change.

Meanwhile, the HP-induced evolution trend of tellurium surface roughness and bulk thickness with respect to the irradiation energy was systematically studied through the atomic force microscopy imaging system. It can be seen from Figure S1c that regardless of the HP irradiation power, the thickness of the tellurium flake area principally showed a thinning trend, accompanied by a slight but monotonic increase in the surface roughness (Figure S1d). It should be noted that the high-power irradiation process gave rise to obvious damage to the sample flake under optical microscope observation; thus, the flake component that did not disappear from the substrate was chosen for an AFM study comparison.

**Figure 3:**
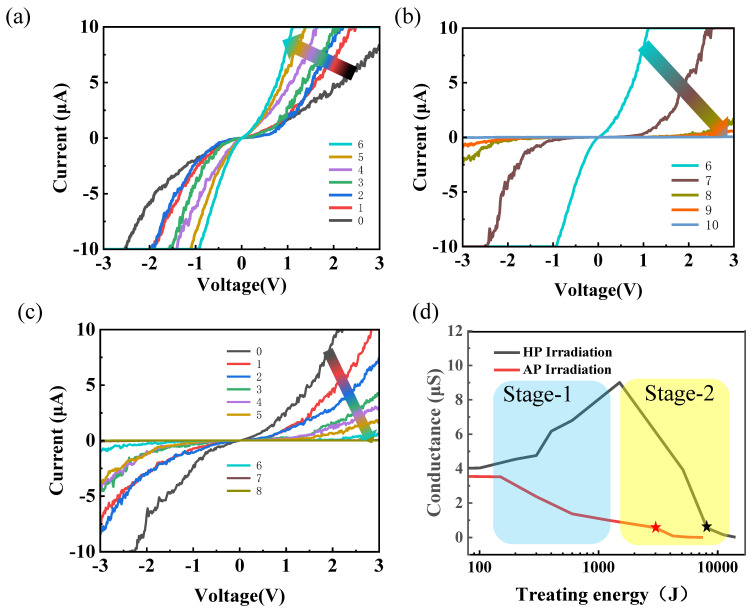
CAFM contact mode probed IV curves of the tellurium flake with HP treatment for stage-1 (**a**) and stage-2 (**b**); (**c**) CAFM contact mode probed IV curves of the tellurium flake under AP treatment. (**d**) The evolution diagram of tellurium conductance with HP and AP irradiation treatment. (The arrows in (**a**–**c**) represent the trend of current changes. The stars in (**d**) indicates the treating energy corresponding to an order of magnitude decrease in conductance).

Though the tellurium crystal flake did not undergo a structural phase transformation or chemical change under HP treatment, the broken lattice causing the flake crystal to be destroyed by the increased irradiation energy is unavoidable. As tellurium is well known for its electronic properties, it is worth paying attention to the evolution of electronic properties under HP irradiation. Here, we transferred another tellurium flake onto a gold electrode substrate, and based on the contact mode of conductive atomic force microscopy (CAFM), the tunnelling current was scanned through the contact bias between the conductive tip and the tellurium flake to reveal the electronic properties and band gap evolution relationship under plasma irradiation [20]. Figure 3a,b give the CAFM contact mode probed IV curves of the tellurium flake under HP treatment with increasing irradiation energies. Surprisingly, the effect of HP irradiation bombardment on the tellurium lattice does not show a monotonous decreasing trend. In the process of HP irradiation with relatively low power and a shorter duration (accumulated in the low-irradiation-energy region), the conductivity of the tellurium flake shows a monotonous rising trend. When the irradiation power further increases, the tunneling current begins to decrease rapidly (Figure 3b). On the other hand, it is worth noting that Faisal Shahzad et al. also significantly improved the electron field emission performance of tellurium nanorod arrays grown perpendicular to a substrate (dropping from 6.22 V/μm to 3.25 V/μm) by HP irradiation treatment, and the authors attributed this improvement to the fact that HP produces numerous abrupt tips, which increase the emission current density and thereby enhances the conductive properties [32]. In this work, the optical images clearly indicate that the tellurium crystal flake surface structure was not damaged by HP irradiation energy accumulation at relatively low power (see the optical images for the first six HP treatments in Figure 2b). As the HP irradiation process is equal to the hydrogen ion implantation effect, it is inferred that such ion implantation in the low-energy region may cause an effective charge doping effect rather than breaking the structural lattice, resulting in an increase in its conductivity. As a consequence, the HP treatment with low energy shows an overall increase in tunnel current.

To confirm this viewpoint, we parallelly used inert atom argon as a plasma gas source to irradiate the tellurium crystal flake in a similar way to that of using HP. As can be seen in Figure S2a, similar to the HP treatment of the tellurium surface, in the low-irradiation-energy region, its surface structure still does not exhibit obvious changes (Figure S2a 0–4). As the AP irradiation treatment power and energy increase, more and more areas on its surface begin to suffer structural damage (Figure S2a 5–8). It is worth noting that during the entire irradiation process, the three intrinsic Raman feature peaks of tellurium (A_1_, E_1_, and E_2_) did not change significantly (i.e., sharpening or broadening of the FWHM; blue- or red-shift of the peak positions), and also no additional new Raman vibration peaks appeared (Figure S2b), which means that even when using high-energy plasma irradiation, it destroyed the microstructure of the flake by breaking its crystal lattice but did not give rise to a new structural phase transformation or chemical change. The AP treatment did not cause changes in Raman features, which is very similar to the HP irradiation effect, as discussed earlier. However, the AP irradiation-induced tunnel current response of the tellurium crystal flake based on the contact bias scanned between the conductive tip and the tellurium surface not only shows a monotonic decreasing trend in the later stage of irradiation with high power, but the tunnel current also directly starts to show a dramatic decreasing trend at the early stage with low-power irradiation (Figure 3c).

To gain a more intuitive understanding of the above-analyzed results, the evolution of tellurium conductance with HP and AP irradiation treatments is shown in Figure 3d. As the treatment energy increases in the low-power region (see the blue layer region in Figure 3d, defined as stage-1 of treatment), the conductance of the HP-irradiated tellurium flake increases significantly. As a comparison, it is very clear to see that even in the stage-1 treatment region, the conductance of the AP-irradiated tellurium flake shows a dramatic decreasing trend. This may be due to the fact that Ar is a much heavier element, and even with lower-power AP treatment, the bombardment effect on the tellurium lattice is still strong. That is to say, compared with HP, AP treatment is more effective in breaking the crystal lattice for tellurium flakes, even in the low-power-based stage-1 irradiation region. On the other hand, the element Ar is more neutral than H; thus, the AP treatment cannot easily provide a charge doping effect that is similar to that of the HP treatment for the tellurium flake. This infers that the AP treatment only gives rise to the bombardment effect to break the crystal lattice but does not have a compensated positive impact on its conductivity properties by charge doping. Furthermore, compared with HP, AP treatment requires less irradiation energy to decrease the conductivity of a pristine flake by one order (see the red and black stars shown in Figure 3d), which could also be attributed to the fact that AP treatment gives a “heavy element” bombardment effect on a tellurium flake compared to that of HP treatment.

**Figure 4:**
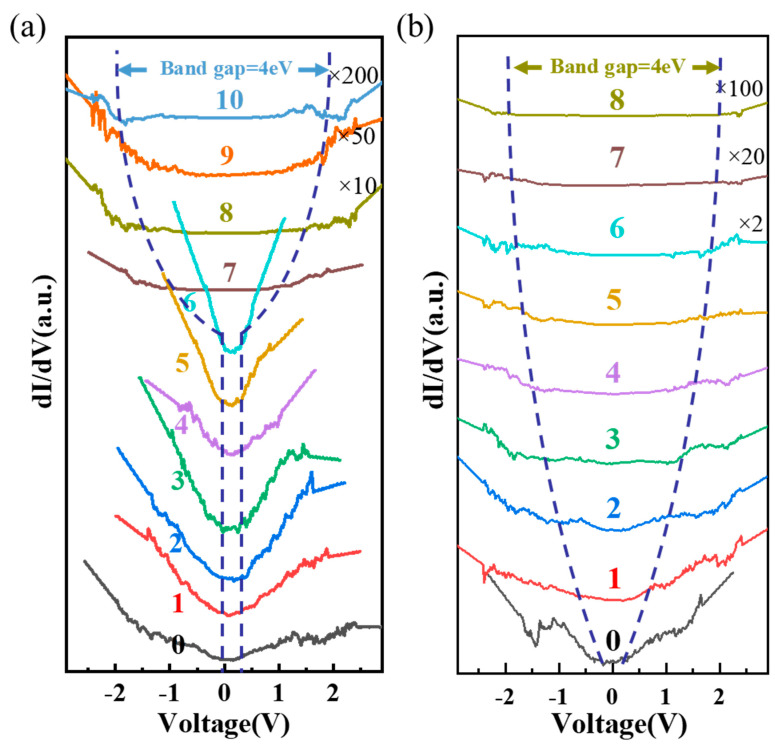
The STS spectra for the HP and AP irradiation-treated tellurium flakes: (**a**) HP irradiation treatment and (**b**) AP irradiation treatment (the distance between the dashed curves in each of the figures schematically shows the approximate trend in the band gap evolution).

In addition to studying the plasma treatment-modulated transport properties of a tellurium crystal flake through measuring the tunnel current (I) based on the contact bias (V) scanning between the conductive tip and the tellurium flake, the evolution of the band gap of the tellurium crystal flake with respect to the plasma treatment was also studied. In fact, the slope of the I–V curve (dI/dV) at each voltage (V) corresponds to the electron density (LDOS) of states at the tip-probed position of the sample. This means that using scanning tunneling spectroscopy (STS) on the basis of dI/dV − V, the zero LDOS region in the STS spectrum can be used to reflect the band gap of the conductive tip-probed tellurium flake. Figure 4 shows the STS spectra for the HP and AP irradiation-treated tellurium flakes, respectively (the STS spectra were derived from the smoothed I–V curves from Figure 3). The evolution of the tellurium flake band gap with respect to the HP irradiation treatment is schematically shown in Figure 4a. It can be seen by the 0th curve in Figure 4a that the band gap is about 0.35 eV for the pristine tellurium, which is close to the band gap of the originally synthesized tellurium powder derived from infrared absorption fitting (Figure 1d). It is clear to see that the corresponding band gap of the tellurium flake during the stage-1 treatment does not show an observable change after each HP irradiation (1st–6th curves in Figure 4a), while starting from the 7th irradiation with relatively high energy (defined as stage-2 treatment, as can be seen in the red layer region in Figure 3d), the band gap gradually enlarges with respect to the HP irradiation treatment. This further corroborates that the HP irradiation in the stage-1 treatment does not strongly influence the crystal structure and, thus, keeps the band gap the same as that of the pristine state, and hence, the HP treatment-induced hydrogen ion charge doping plays the main role in enhancing its transport performance, which is consistent with the previously discussed result during the stage-1 treatment. For stage-2 HP irradiation treatment, the band gap of the tellurium flake becomes enlarged step by step. This is probably ascribed to the high-energy-irradiation-caused heavily broken crystal lattice, and thus, the formation of local defects leads to the flake becoming more amorphized and presenting a larger band gap. The larger band gap of the high-power HP-induced broken-lattice tellurium flake is another factor causing the rapid degradation of the transport performance. For comparison, the effect of the AP treatment on the tellurium flake does not follow similar trend to that of the HP treatment. As shown in Figure 4b, the evolution of the dI/dV spectra with a bias voltage clearly indicates that the band gap of the tellurium flake is monotonically enlarged for both irradiation treatments of stage-1 and stage-2, which means that from the initial stage of the AP treatment, the defects caused by the destruction of its internal crystal lattice begin to affect the enlargement of its band gap. Therefore, even for the low-power-based stage-1 AP treatment irradiation, it directly gives rise to the formation of local defects and an enlargement of the band gap, such that the dual effects synergistically cause a dramatic degradation in the transport performance.

## 4. Conclusions

In this study, we comprehensively investigated the evolution of morphology, lattice vibration, conductivity, and band gap of tellurium crystal flakes under both HP and AP irradiation treatments. Our findings confirm that the “gentle” treatment during stage-1 HP irradiation with low power does not significantly alter the band gap or macrostructure of tellurium flakes; however, it notably enhances their conductivity. Conversely, when the irradiation energy surpasses a certain threshold, the band gap widens, and the flake’s macrostructure undergoes a gradual degradation due to lattice breaking, thereby compromising its transport properties. In contrast, argon (a natural and heavy element)-based plasma treatment of tellurium flakes, even at low power during stage-1 irradiation, fails to enhance their conductivity. This study not only characterizes the effects of HP and AP plasma irradiation treatments on the crystal structure of tellurium flakes but also directly examines the evolution of their electronic properties and corresponding trends in band gap changes following plasma treatment. Moreover, we identified a beneficial modulation window that significantly enhances the electronic performance of tellurium flakes through HP irradiation treatment at low power. Our work contributes to a deeper understanding of the effects of plasma irradiation on tellurium crystal flakes and opens up new avenues for electronic studies on tellurium and other 2D materials.

## Data Availability

The datasets generated during the study are available from the corresponding author upon request.

## Supplementary Materials

The following supporting information can be downloaded at: https://www.mdpi.com/article/10.3390/nano14090750/s1, Figure S1: Atomic force microscope characterization of tellurium flakes irradiated by HP; Figure S2: Characterization of tellurium flakes irradiated by HP.
