# Supplementary material for: Evolution of the Electronic Properties of Tellurium Crystals with Plasma Irradiation Treatment
Source: Nanomaterials (Basel). 2024 Apr 25;14(9):750. doi: 10.3390/nano14090750 (PMC11085414; doi:10.3390/nano14090750)
Supplement: Supplementary file 1 [file nanomaterials-14-00750-s001.zip › nanomaterials-2960346-supplementary.pdf]

Supplementary Materials

The Electronic Properties Evolution of Tellurium Crystals with Plasma Irradiation Treatment

Congzhi Bi[1,2], Tianyu Wu[2,3], Jingjing Shao[2], Pengtao Jing[2], Hai Xu[2], Jilian Xu[2], Wenxi Guo[1,*], Yufei Liu[4,*], and Da Zhan[2,*]

[1] Department of Physics, College of Physical Science and Technology, Research Institution for Biomimetics and Soft Matter, Xiamen University, Xiamen 361005, China; 19820211153715@stu.xmu.edu.cn (C.B.);

[2] State Key Laboratory of Luminescence and Applications, Changchun Institute of Optics, Fine Mechanics and Physics, Chinese Academy of Science, Changchun 130033, China; 1130907780@qq.com (J.S.); jingpt@ciomp.ac.cn (P.J.); xuhai@ ciomp.ac.cn(H.X.); xujl@ ciomp.ac.cn(J.X.);

[3] College of science, Beihua University, Jilin 132000, China; wutianyu0308@126.com (T.W.)

[4] Key Laboratory of Optoelectronic Technology & Systems (Chongqing University), Ministry of Education, Chongqing 400044, China; yufei.liu@cqu.edu.cn (Y.L.)

[*] Correspondence: zhanda@ciomp.ac.cn (D.Z.); yufei.liu@cqu.edu.cn (Y.L.); wxguo@xmu.edu.cn (W.G.)

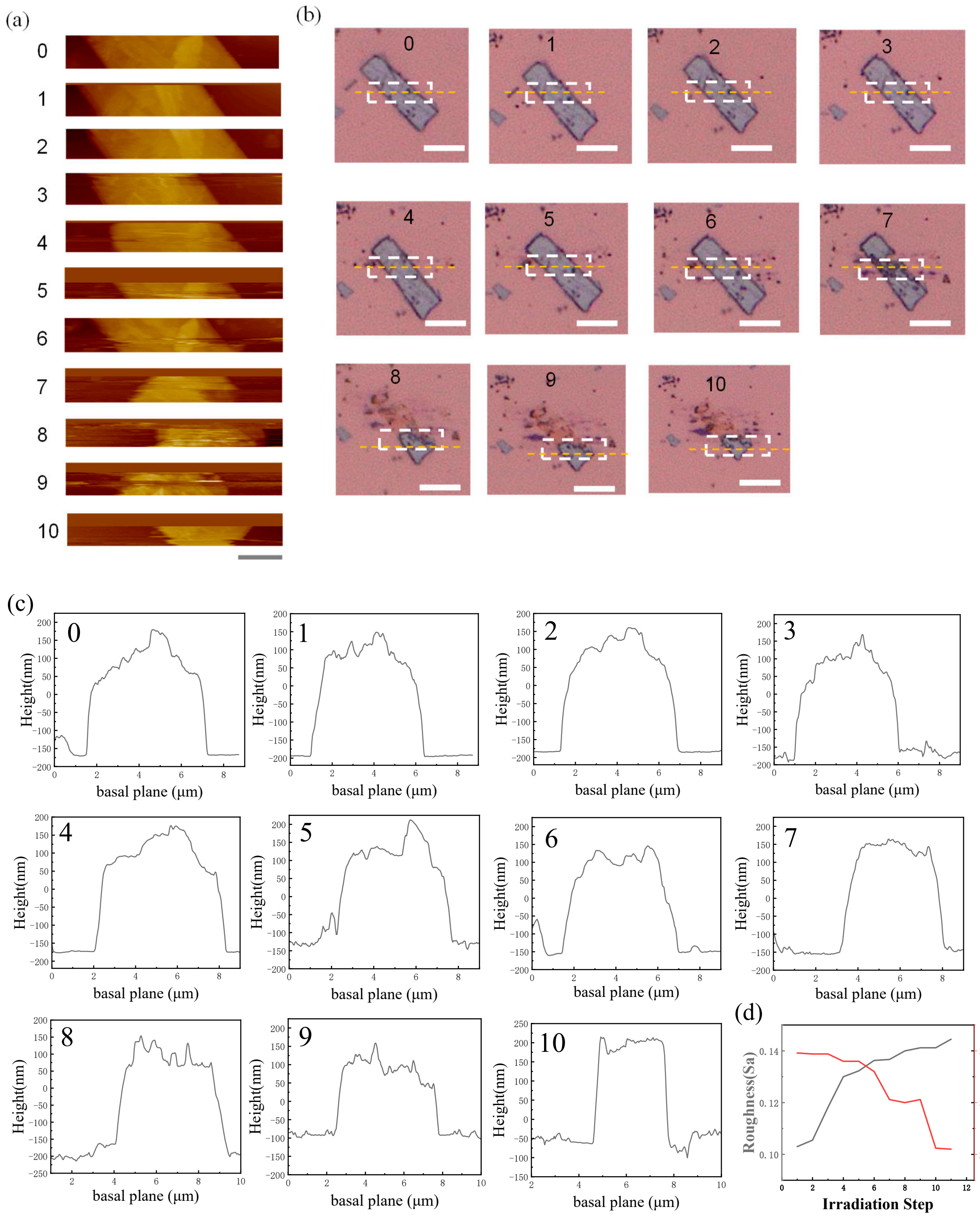

Figure S1. Atomic force microscope characterization of tellurium flake irradiated by HP. (a) atomic force microscope images, the scale bar is 1um; (b) the optical microscope images show the corresponding scanned AFM region by the white dashed square (the scale bar is 3um for each image), and the orange dashed line in each image corresponds to the cross section height profile that shown in (c), the number in the upper corner represents the irradiation steps; (d) the evolution of surface roughness and height of tellurium flakes with irradiation step.

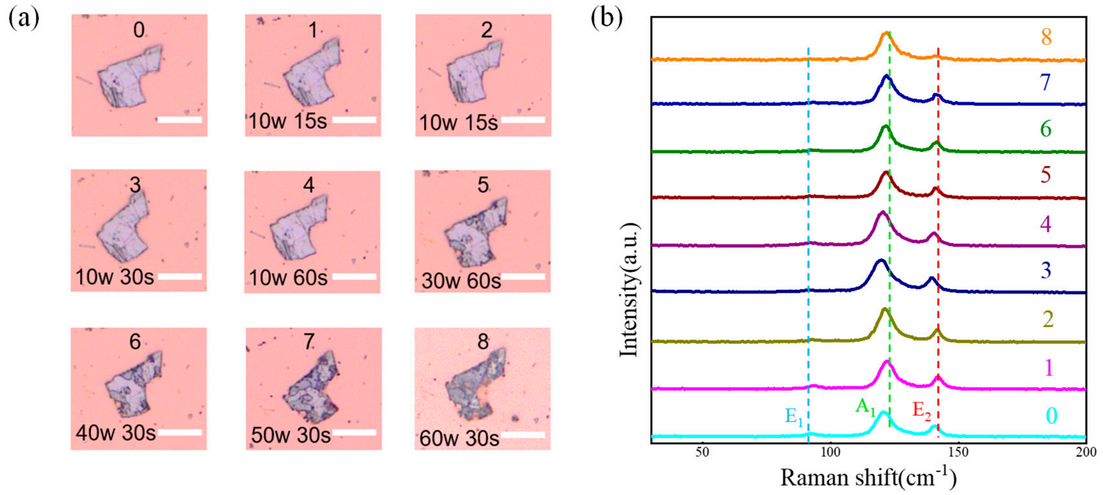

Figure S2. Characterization of tellurium flakes irradiated by AP. (a) The optical microscope images with the scale bar of 5um (the irradiation power and duration are marked accordingly for each image); (b) Raman spectra of the tellurium flake with successive AP irradiation treatment.
